# Supplementary material for: Ehlers–Danlos Syndrome and Hypermobility Syndrome Compared with Other Common Chronic Pain Diagnoses—A Study from the Swedish Quality Registry for Pain Rehabilitation
Source: J Clin Med. 2020 Jul 7;9(7):2143. doi: 10.3390/jcm9072143 (PMC7408708; doi:10.3390/jcm9072143)
Supplement: Supplementary file 1 [file jcm-09-02143-s001.pdf]

Supplementary material

Table S1: The Villefranche criteria for major EDS subtypes 1

Subtype	Major criteria	Minor criteria
Classical	Skin hyperextensibility<br>Widened atrophic scars<br>Joint hypermobility	Smooth, velvety skin<br>Molluscoid pseudotumors<br>Subcutaneous spheroids<br>Complications of joint hypermobility<br>Muscle hypotonia, motor delay<br>Easy bruising<br>Manifestations of tissue extensibility and fragility<br>Surgical complications<br>Positive family history
Hypermobility	Hyperextensible and/or smooth, velvety skin<br>Generalized joint hypermobility	Recurring joint dislocations<br>Chronic joint/limb pain<br>Positive family history
Vascular	Thin, translucent skin<br>Arterial/intestinal/uterine fragility or rupture<br>Extensive bruising<br>Characteristic facial appearance	Acrogeria<br>Hypermobility of small joints<br>Tendon and muscle rupture<br>Talipes equinovarus<br>Early-onset varicose veins<br>Arteriovenous, carotid-cavernous sinus fistula<br>Pneumothorax/pneumohemothorax<br>Gingival recessions<br>Positive family history, sudden death in a close relative
Kyphoscoliosis	Generalized joint hypermobility<br>Congenital hypotonia<br>Congenital and progressive scoliosis<br>Scleral fragility and rupture of the ocular globe	Tissue fragility, including atrophic scars<br>Easy bruising<br>Arterial rupture<br>Marfanoid habitus<br>Microcornea<br>Osteopenia/porosis<br>Positive family history
Arthrochalasia	Generalized joint hypermobility with recurrent subluxations<br>Congenital bilateral hip dislocation	Skin hyperextensibility<br>Tissue fragility, including atrophic scars<br>Easy bruising<br>Hypotonia<br>Kyphoscoliosis<br>Osteopenia/porosis
Dermatosparaxis type	Severe skin fragility<br>Sagging, redundant skin	Soft, doughy skin texture<br>Easy bruising<br>Premature rupture of fetal membranes<br>Large hernias (umbilical, inguinal)

The presence of one or more major criteria is either necessary for clinical diagnosis or highly indicative and warrants laboratory confirmation whenever possible. A minor criterion is a sign of lesser diagnostic specificity. The presence of one or more minor criteria contributes to the diagnosis of a specific type of EDS. However, in the absence of major criteria they are not sufficient to establish the diagnosis.

Table S2: The revised (Brighton 1998) criteria for the diagnosis of benign joint hypermobility syndrome [2]

**Major criteria**	
	Beighton score ≥ 4<br>Arthralgia for longer than 3 months in 4 or more joints
**Minor criteria**	
	Beighton scores 1-3<br>Arthralgia in 1 to 3 joints or back pain or spondylosis, spondylolysis and/or spondylolisthesis<br>Dislocation in more than one joint or in one joint on more than one occasion<br>Three or more soft tissue lesions (eg, epicondylitis, tenosynovitis, bursitis)<br>Marfanoid habitus<br>Abnormal skin: striae, hyperextensibility, thin skin, papyraceous scarring<br>Eye signs: drooping eyelids, myopia, or antimongoloid slant<br>Varicose veins, hernia, or uterine or rectal prolapse

Joint hypermobility syndrome is diagnosed in the presence of two major criteria; one major criterion plus two minor criteria; or four minor criteria. Two minor criteria will suffice where there is an unequivocally affected first degree relative.

[1] Adapted from Beighton P, De Paepe A, Steinmann B, Tsipouras P, Wenstrup RJ. Ehlers-Danlos syndromes: revised nosology, Villefranche, 1997. *American Journal of Medical Genetics*. 1998;77:31–37.

[2] Adapted from Grahame R, Bird HA, Child A. The revised (Brighton 1998) criteria for the diagnosis of benign joint hypermobility syndrome (BJHS). J Rheumatol, 2000; 27:1777–1779
